# Indications, Techniques, and Outcomes of Robot-Assisted Insular Stereo-Electro-Encephalography: A Review

**DOI:** 10.3389/fneur.2020.01033

**Published:** 2020-09-17

**Authors:** Amaury De Barros, Julien Francisco Zaldivar-Jolissaint, Dominique Hoffmann, Anne-Sophie Job-Chapron, Lorella Minotti, Philippe Kahane, Emmanuel De Schlichting, Stephan Chabardès

**Affiliations:** ^1^Department of Neurosurgery, Toulouse University Hospital, Toulouse, France; ^2^CHU Grenoble Alpes, Clinical University of Neurosurgery, Grenoble, France; ^3^CHU Grenoble Alpes, Clinical University of Neurology, Grenoble, France

**Keywords:** epilepsy, SEEG (stereoelectroencephalography), stereotaxic, epilepsy surgery planning, robot-assisted surgery (RAS)/computer assisted surgery (CAS)

## Abstract

Stereo-electro-encephalography (SEEG) is an invasive, surgical, and electrophysiological method for three-dimensional registration and mapping of seizure activity in drug-resistant epilepsy. It allows the accurate analysis of spatio-temporal seizure activity by multiple intraparenchymal depth electrodes. The technique requires rigorous non-invasive pre-SEEG evaluation (clinical, video-EEG, and neuroimaging investigations) in order to plan the insertion of the SEEG electrodes with minimal risk and maximal recording accuracy. The resulting recordings are used to precisely define the surgical limits of resection of the epileptogenic zone in relation to adjacent eloquent structures. Since the initial description of the technique by Talairach and Bancaud in the 1950's, several techniques of electrode insertion have been used with accuracy and relatively few complications. In the last decade, robot-assisted surgery has emerged as a safe, accurate, and time-saving electrode insertion technique due to its unparalleled potential for orthogonal and oblique insertion trajectories, guided by rigorous computer-assisted planning. SEEG exploration of the insular cortex remains difficult due to its anatomical location, hidden by the temporal and frontoparietal opercula. Furthermore, the close vicinity of Sylvian vessels makes surgical electrode insertion challenging. Some epilepsy surgery teams remain cautious about insular exploration due to the potential of neurovascular injury. However, several authors have published encouraging results regarding the technique's accuracy and safety in both children and adults. We will review the indications, techniques, and outcomes of insular SEEG exploration with emphasis on robot-assisted implantation.

## Introduction

The insular cortex is anatomically located deep inside the lateral sulcus, enclosed and covered by the frontoparietal and temporal opercula. Its hidden location inspired Gray to name this deep cortical structure the “Island of Reil,” in tribute to the seminal description of the insula by Christian Reil (1). The neurofunctional role of the insula remained poorly understood, and its role in brain network organization and connectivity was underestimated until pioneers like Penfield and Faulk in Montreal (2), and Guillaume and Mazars in Paris (3), who both described the involvement of the insula in refractory epilepsy using electrocorticography during anterior temporal lobectomy surgeries. But before the advent of modern presurgical investigations and microsurgical techniques, insular surgery in addition to anterior temporal resection was considered at risk with high morbidity and poor effectiveness to increase epileptic control (4). This explains why the issue of insular investigation and resection entered a silent period of almost four decades, with the exception of very few studies of lesion cases.

A major breakthrough occurred in 2000, when Isnard et al. using stereo-electro-encephalography (SEEG), demonstrated that some failures of temporal lobectomy could be related to seizures originating in the insular cortex (5). Since then, the role of the insula in surgically focal epilepsy has been extensively investigated (6), showing first that insular (or insulo-opercular) cortex epilepsy may mimic temporal, frontal, or parietal epilepsy, and second that the insula can be part of widely extended epileptogenic zones such as in the case of temporal “plus” epilepsies (7). Overall, the complexity of focal epilepsies involving the insula makes the precise determination of the ictal topographical culprit of paramount importance, especially when a surgical treatment is envisioned. In this context, SEEG recordings are especially well-suited to giving access directly to deep brain structures that cannot be recorded using subdural grids or strips. Recent developments in imaging, computer-assisted planning (particularly image fusion algorithms and planning software), and robotic assistance currently allow safer and more accurate electrode insertion for performing electrophysiological recordings. Still, robotics has gained interest in the epilepsy surgery field due to their reliable, reproducible, safe, accurate, and time-saving electrode insertion potential and their versatility for orthogonal and oblique electrode insertions.

We will review here the indications, techniques and outcomes of insular SEEG exploration, emphasizing the current role of computational-image guidance and robot-assistance.

## Indications for Insular SEEG

As a general rule, SEEG indications in insular epilepsy do not significantly differ from those proposed for other drug-resistant focal epilepsy, the clinical data—corroborated by ictal scalp-EEG and neuroimaging findings—being among the most important features. In the specific context where the insula can be part of the epileptogenic zone, two situations must be distinguished: either (i) the seizures are suspected to originate from the insula before they spread to other cortical areas (including the contralateral insula), therefore mimicking perisylvian, frontal, temporal, and even parietal seizures (8) or (ii) the insula is part of a more diffuse epileptogenic network as in temporo-insular epilepsy, which is the most frequent form of temporal “plus” epilepsies (9). In these scenarios, auras are of paramount importance, such as painful somatosensory sensations (10), olfactory, gustatory, auditory, and vestibular manifestations (11); or breathlessness, laryngeal discomfort, and perioral paresthesiae (12). Other clinical features, such as a reflex component of the seizures (13), or an ictal bradycardia/asystole (14–16) can also suggest an insular involvement. For Nguyen and colleagues (17), the insula should be explored whenever a clinical pattern other than the one expected to emerge from the ictal onset zone comes to the fore, especially in MRI negative cases. The combination of some of the ‘‘specific'' insular (or insulo-opercular) fingerprints described above, as well as their occurrence with hypermotor behavior (18), somato-motor signs, temporal-like automatism or spasm-like behavior, should lead to consideration of the insula (or insulo-opercular complex) as a possible seizure onset zone.

In addition to these clinical cues, SEEG should include an insular exploration when (i) there exists an insular lesion likely responsible for the seizure, in order to precisely delineate the borders of the resection, to perform a functional mapping (e.g., the dominant hemisphere for language) or to apply radiofrequency thermocoagulation; (ii) there is a cortical anomaly on standard MRI imaging in the close vicinity of the insula (e.g., supra- or infra-sylvian operculum, posterior part of the orbito-frontal cortex); or (iii) there is a suspicion of insular involvement in EEG-HD, MEG, fMRI, or PET studies (19).

### Implantation Strategy in Suspected Insular Cases

The complexity of the insular cortex and the many forms of insular lobe seizures make SEEG implantation a challenge whenever insular epilepsy is suspected. The basic principle, however, remains the same as for any SEEG study: the number, targets, and trajectories of insular as well as extra-insular electrodes being personalized according to patients' specific profiles. While most patients have an epileptogenic zone extending beyond the insula, some may have a very focal seizure onset, which first needs a large insulo-opercular coverage. The best approach therefore combines an oblique approach through the frontal or parietal cortices to allow a larger insular sampling, with a lateral orthogonal trajectory through the fronto-parietal and temporal operculum in order to disentangle the insula from operculum involvement in seizure generation (see below). A bilateral insulo-opercular implantation has to be considered whenever the seizure lateralization is unclear. To better evaluate the extent of the future resection and to exclude any extra-insular onset, seizure spread must also be evaluated, which requires an appropriate sampling of the extra-insular regions to which the insula is closely connected. This extra-insular spread, which occurs often, early, and rapidly after the seizure onset, accounts for the majority of the semiological features and can therefore be anticipated from clinical seizure analysis (frontal vs. perisylvian vs. temporal semiology).

### Robot-Assisted SEEG

#### Historical Perspective and Hardware Development

*Robot* is derived from the root of the Czech word “robota” which means “forced labor.” That resumes the human desire to delegate tiring, difficult, and repetitive tasks to technology. In this way, robot-assisted surgery has been developed in almost the entire field of surgery, including neurosurgery (20, 21). With the exponential development of robotics, stereotactic neurosurgeons rapidly recognized their potential for clinical use.

The first robot-aided neurosurgical procedure (a stereotactic biopsy) was carried out in 1985, making use of an industrial robot (PUMA 200) (22). A few years later, Benabid et al. in Grenoble, France, began to develop dedicated neurosurgical robots for general micro-neurosurgical procedures (using a Surgiscope microscope) and stereotactic procedures (Neuromate).

The Neuromate® robot (Renishaw-mayfield; Nyon, Switzerland) was developed and used successfully for brain biopsies, deep-brain stimulation, and SEEG (23, 24) and approved by the Food and Drug Administration for commercialization, leading to its worldwide diffusion and utilization for stereotactic procedures including SEEG. The ROSA® (Medtech SAS, Zimmer Biomet, Montpellier, France) robotic arm was later developed in the 2000's in France, based on an industrial robotic arm. It differed from the Neuromate in its built-in haptic capabilities, allowing the intuitive mobilization of the arm by the surgeon as an extension of herself. It also possesses a user-friendly graphical interface with a touch-screen monitor that can be used intraoperatively. The ROSA® is additionally more mobile (six movement axes as compared to five for the Neuromate).

The last-born commercial robotic arm is the iSYS1® (iSYS Medizintechnik, Kitzbühel, Austria), a novel miniature robotic arm with four axes of freedom that attaches to a classic three-pin headholder such as the Mayfield clamp (25). Neuromate® and ROSA® offer the possibility to perform both frame-based or frameless techniques while the iSYS1® offers a frameless technique only.

Other robotic arms had been developed over the years but were never used for SEEG or broadly commercialized.

#### Frame-Based Robot-Assisted SEEG Technique

The original Talairach's frame-based technique used an external fixed-grid system coupled with intraoperative teleangiography and ventriculography in order to allow the positioning of orthogonal electrodes in the desired place using two-dimensional imagery (26).

Nowadays, MRI and image fusion with digital subtraction angiography (DSA) have become the gold standard for SEEG pre-operative planning, as they are accurate and reliable.

Using a classical manual Leksell stereotactic frame, several epilepsy centers have described techniques of orthogonal and oblique depth-electrode insertion in the insula with good accuracy and few complications (27–29).

The first automatic robot-assisted techniques followed the same basic principles and used a cranial frame fixed on a robot (24). Robot assistance made oblique trajectories much easier due to robot reliability, versatility (multiple movement axes), and insertion-axis rigidity (30). Several groups have published their experience with frame-based robot-assisted methods (31–34), which have proven to be safe and accurate. Recently, frameless techniques have emerged in order to gain time without compromising accuracy.

#### Frameless Robot-Assisted SEEG Technique

**Figure 1:**
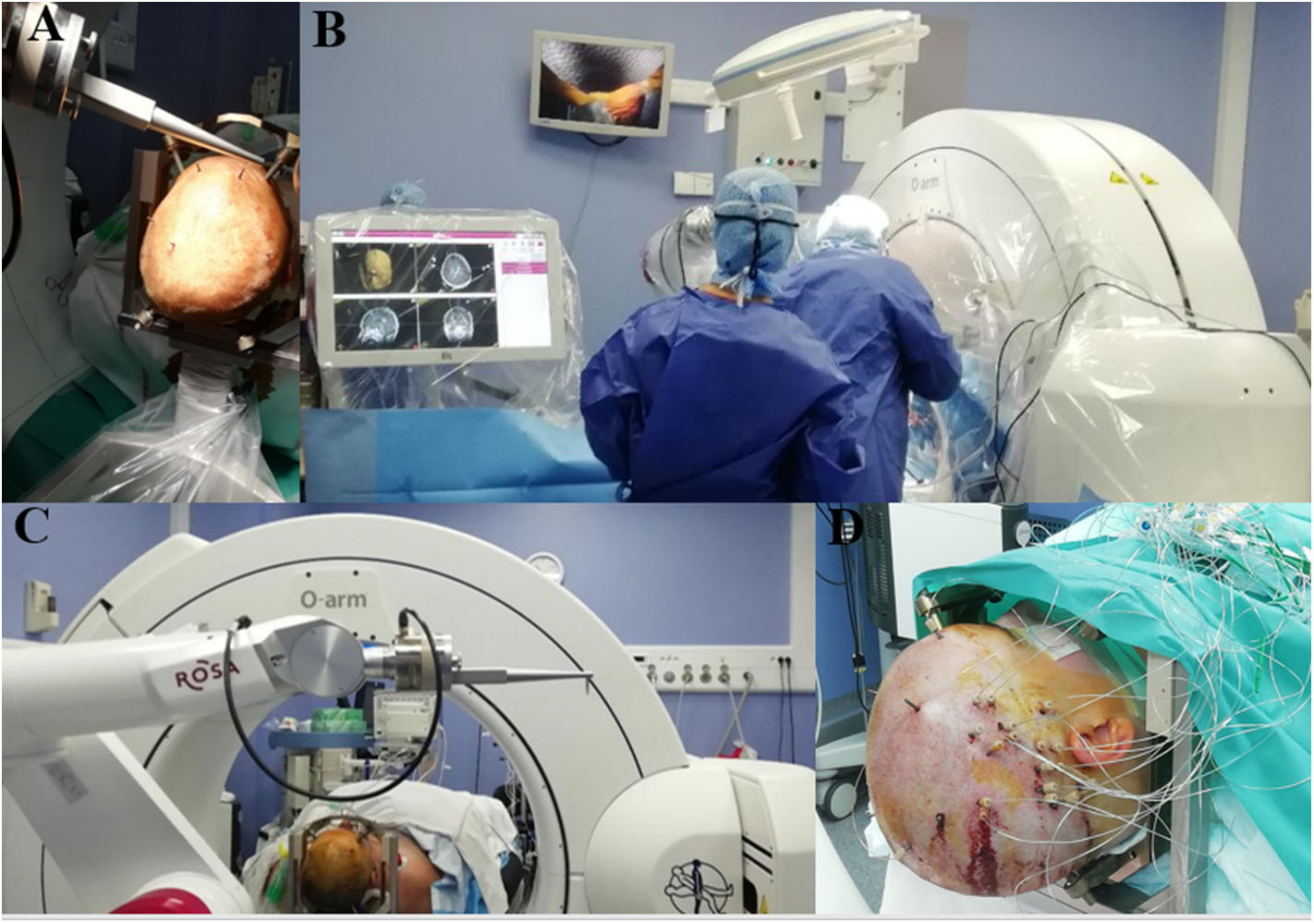
Example of ROSA-robot system in the operating room in Grenoble. **(A)** Five bone fiducials markers anchored in the patient skull in non-coplanar manner. **(B)** ROSA-robot system with sterile touchscreen easy to use interface and intraoperative CT. **(C)** ROSA-robot dedicated bone fiducials markers system for referencing process. **(D)** Immediate postoperative view of a right SEEG.

**Figure 2:**
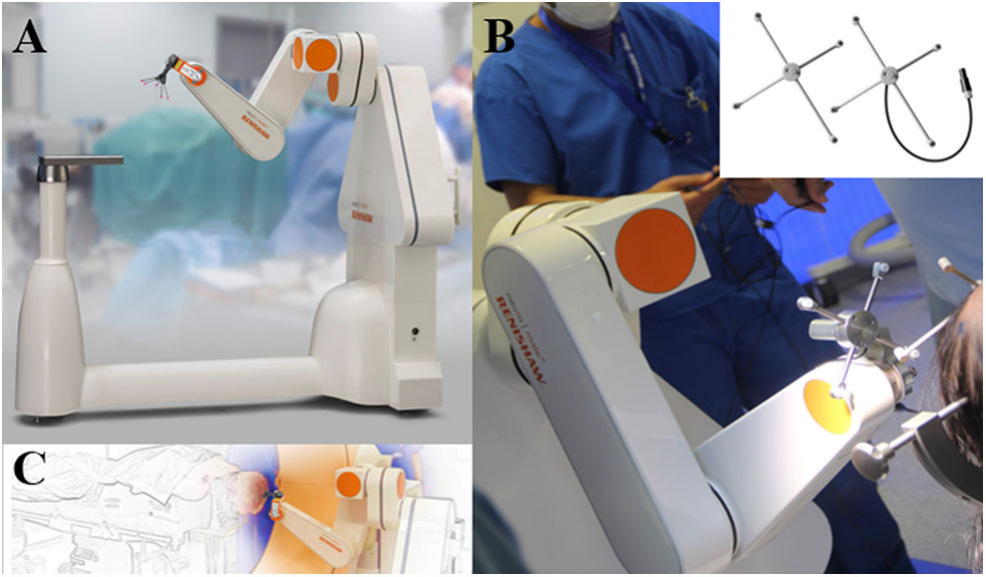
Neuromate-Robot system by Renishaw. **(A,C)** Neurolocate® system used for frameless registration. **(B)** Registration with Ultrasound technology. Images provided by RENISHAW with permission for publication (Renishaw-mayfield; Nyon, Switzerland).

The development of frameless insertion techniques aimed to simplify the process, with gains in time and procedural simplicity. These methods require a referencing step in order to match the patient's anatomy with the radiological images and pre-surgical planning. Laser-based facial referencing is possible with the ROSA® robot. As with current neuronavigation systems, several facial and skull landmarks are collected by the laser system in order to accurately superimpose the 3-D radiological exams on the patient's real anatomy. It is worth noting that using a CT exam for the laser referencing process appears to be much more accurate than using MRI, thus adding an additional step of CT/MRI image fusion (35). In our center, we use bone fiducial markers and intraoperative CT imaging (O-Arm®, Medtronic, Minneapolis, USA) for the referencing process and fuse the images with our MRI target plan and angiography. Trajectories are planned a week before the surgery on the ROSA® software. The resulting trajectories are executed by the ROSA® robot once the referencing accuracy has been carefully checked with the patient's anatomy (Figure 1). This technique was compared to frame-based methods on SEEG in term of complications but not accuracy (36). Our accuracy data published on DBS with sub-millimetric accuracy highlight the precision of this method (37). Dorfer et al. (25) used bone fiducial markers with the iSYS1® robot for SEEG, reporting millimetric accuracy. In Milan, Cardinale et al. (38) used the Neuromate® dedicated fiducial markers (Neurolocate) developed by Renishaw (Mayfield, Nyon, Switzerland) and mounted on the robot arm, rendering the use of bone or skin fiducial markers unnecessary with a gain of time and simplicity. Neuromate® offers another possibility of referencing based on ultrasound registration and is used by other teams (39) (Figure 2).

#### Insular SEEG Electrode Trajectories: Advantages and Limitations

##### Orthogonal approach **(****Figure 3****)**

The first papers describing insular SEEG sampling used an orthogonal trans-opercular approach (5, 40). The entire perysylvian area can be recorded with information from either opercula and insula. However, the orthogonal approach imposes sampling limitations, with few contacts per electrode that are actually in the insular cortex, requiring the multiplication of trajectories with presumed greater vascular risk.

**Figure 3:**
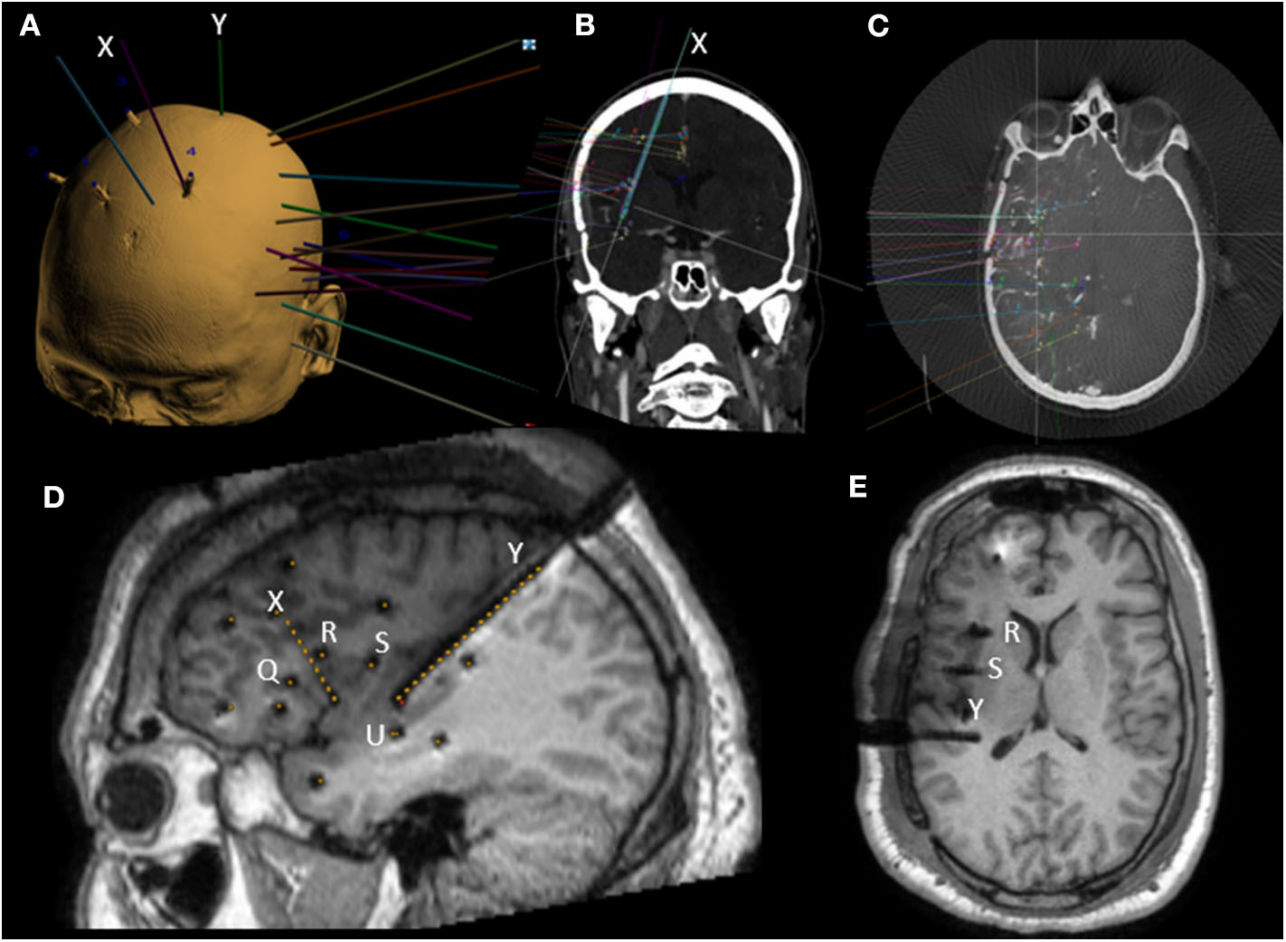
Example of preoperative SEEG planning on ROSA software and postoperative MRI with mixed orthogonal and oblique trajectories. **(A)** 3D view of a left SEEG planning along with bone fiducials markers. **(B)** In plane view along oblique anterior insular (X) trajectory. **(C)** Example of SEEG planning fused with 3D angiography. **(D)** Postoperative sagittal T1 MRI with dark shadows representing electrodes with yellow dots superimposed representing planned trajectories. Note the oblique trajectories (X and Y) and four orthogonal trajectories (Q, R, S, and U). **(E)** Postoperative axial T1 MRI with dark shadows representing electrodes. Note the end location of contacts in the upper insula of orthogonal electrodes (R and S).

##### Oblique approach **(****Figure 3****)**

Oblique approaches were developed through frontal and parietal entry points, in order to allow more extensive insular sampling while also limiting the vascular risks (30). A larger number of insular contacts are achieved through a less vascular intraparenchymal route. Oblique bone drilling, however, represents a technical challenge because of bone ripping, and the planned trajectories have to be longer. Both of these factors can result in target point inaccuracy. The second main pitfall is the occasional proximity of the entry point to the superior sagittal sinus, leading to possible transection of venous Trolard's lakes, but this problem is encountered in practice only exceptionally.

##### Posterior parasagittal trans-insular approach

As with the oblique approach, the posterior approach allows insular sampling that is widely distributed, and electrodes can reach the amygdala (28). However, when the implantation scheme is not planned specifically for posterior implantation, the multiplicity of electrodes placed in standard SEEG makes the use of this trajectory challenging as the posterior parieto-occipital entry point can be difficult to access, even with robotic assistance. Furthermore, as with the oblique approach, the length of the trajectory can be a factor for inaccuracy. Finally, the Sylvian cistern can be transected in the posterior part of the peri-insular sulcus.

##### Insular SEEG accuracy

Few studies exist on the accuracy of SEEG electrode implantation relative to planned trajectories, and the specific literature on insular trajectories is even more scarce. Table 1 summarizes the different studies published and available at the date of this review.

**Table 1:** Relevant literature about insular robot-assisted SEEG accuracy.

**Epilepsy center/study**	**Patients**	**Method**	**Frameless referencing process**	**Number of procedures/number of electrodes/number of insular electrodes**	**Error measure**	**Entry point**	**Target point**
Milan (Italy) Cardinale et al. (33) Cardinale et al. (38)	A and P	Neuromate frameless vs. neuromate frame-based vs. conventional frame-based method	Neurolocate	8/127/NR vs. 81/1,050/NR vs. 37/517/NR	Euclidean distance	All electrodes 0.59 vs. 0.78 vs. 1.43	All electrodes 1.49 vs. 1.77 vs. 2.69
Cleveland (USA) Gonzalez-Martinez et al. (41)	A and P	Rosa Frameless vs. conventional frame-based method	Laser-based facial scanning	101/1,245/NS vs. 103/1,367/NR	Euclidean distance	All electrodes 1.2 vs. 1.1	All electrodes 1.7 vs. NR
Vienna (Austria) Dorfer et al. (25)	A and P	iSys1 frameless	6 bone fiducial markers	16/93/13 5 of the 16/31/NR with K-wire	Lateral deviation	All electrodes 1.3 1.54–1.18 with K-wire^a^	All electrodes 1.5 1.82–1.66 with K-wire^a^
Strasbourg (France) Ollivier et al. (42)	A and P	ROSA frameless	Laser-based facial scanning	66/901/NR	Euclidean distance	All electrodes Orthogonal:1.39 Oblique:1.63	All electrodes Orthogonal:2.61 Oblique:3.29
Roma (Italy) De Benedictis et al. (43)	P	ROSA frameless	Laser-based facial scanning	36/386/NR	Euclidean distance	All electrodes 1.50	All electrodes 1.96
Barcelona (Spain) Candela-Canto et al. (39)	P	Neuromate	Ultrasound co-registration	14/164/NR	Planning software (formula not described)	All electrodes 1.57	All electrodes 1.77
Lyon (France) Bourdillon et al. (32)	A and P	Neuromate Frame-based vs. conventional frame-based method	/	50/565/96 vs. 50/628/NR	Euclidean distance	NR	All electrodes 1.15 vs. 4.00
Great ormond street hospital London (UK) Sharma et al. (44)	P	Neuromate frame-based vs. optical neuro-navigation (ON)	/	20 (14 robot; 6 ON)/218/NS	Euclidean distance	All electrodes Robot: 0.71 ON: 5.5	All electrodes Robot: 1.07 ON: 4.5
London (Canada) Bottan et al. (31)	A	Neuromate frame-based (+ 1 bone fiducial marker)	/	41/98/98	Euclidean distance	**Insular electrodes** Orthogonal: 1.5 Oblique: 1.5 Parasagittal: 1.3	**Insular electrodes** Orthogonal: 1.9 Oblique: 2.4 Parasagittal: 1.5
Frankfurt (Germany) Spyrantis et al. (35)	A	ROSA frame-based vs. ROSA frameless	CT laser-based facial scanning vs. MRI laser-based facial scanning	19/171/15 CT-frame: 4/49/8 CT- laser: 7/60/1 3T MRI-laser: 7/56/6 1.5T MRI-laser: 1/6/0	Euclidean distance	**Insular electrodes:** CT-frame: 0.88 3T MRI-laser: 3.36 **All electrodes:** CT-frame: 0.86 CT-laser: 1.85 3T MRI-laser: 3.02 1.5T MRI-laser: 0.97	**Insular electrodes:** CT-frame: 1.99 3T MRI-laser: 4.07 **All electrodes:** CT-frame: 2.28 CT-laser : 2.41 3T MRI-laser: 3.51 1.5T MRI-laser: 1.71
Munt Sinaï; New York (USA) Iordanou et al. (45)		ROSA frameless	CT laser-based facial scanning or bone fiducials	25/319/NR	Lateral deviation (LD) and depth error (DE)	**All electrodes: Oblique electrodes** LD:1.76 **Orthogonal electrodes** LD:1.32	**All electrodes: Oblique electrodes** LD: 2.05 DE: 2.32 **Orthogonal electrodes:** LD: 1.45 DE: 2.33

*A, Adults; EP, Entry point error; NR, Not reported; P, Pediatric; TP, Target point error*.

a *The authors developed a technique for skull drilling based on pre-drill with a K-wire with an improved accuracy*.

Different methods have been used to calculate trajectory accuracy. Most authors have used the 3D Euclidean distance for assessing electrode divergence from the initial target plan in the three spatial planes at both the entry point and the final target using the formula

$$\begin{array}{l} Ed\;\left(a,b\right)=\sqrt{{\left(Xa-Xb\right)}^{2}+{\left(Ya-Yb\right)}^{2}+{\left(Za-Zb\right)}^{2}} \end{array}$$

It is noteworthy that the lateral deviation, the radial error in a given plane, gave shorter distances compared to the Euclidean distance as emphasized by Ho et al. (46) with, at the target point, a lateral deviation estimate of 1.75 mm compared to 3.39 mm for the Euclidean distance (without insular electrodes).

Globally, entry-point errors range from 0.5 to 1.5 mm with either frame-based or frameless robot-assisted methods. Laser-based facial referencing with MRI appears to have a larger error margin (35). Entry-point errors seem to be dependent on the temporal pole position during surgery, the thickness of the scalp and the angle of the trajectory relative to the tangential plane of the skull (33). Inaccuracies at the entry point and distance to the target logically have a negative impact on target accuracy. An angle > 30° between the skull and the trajectory can lead to significant target deviations (45). Globally, target errors are in a range from 1.0 to 3.0 mm with 0.5 to 1.5 mm of difference between oblique and orthogonal trajectories. For comparison, with conventional frame-based SEEG (Tables 1, 2), target errors range from 2.0 to 4.0 mm. Neuronavigation systems have smaller accurate results in accordance with Vakharia et al. (51).

**Table 2:** Relevant literature about non robot-assisted SEEG accuracy for insular trajectories.

**Epilepsy center/study**	**Patients**	**Technique**	**Number of patient/number of electrodes/number of insular electrodes**	**Error measure**	**EP (mm)**	**TP (mm)**
National hospital for neurology and neurosurgery; London (UK) Nowell et al. (47)	A	Frameless neuronavigation	22/187/15	Lateral deviation	NR	**All:** 3.66 **Insula:** 2.83
Rouen (France) Gilard et al. (48)	A and P	Conventional frame-based	10/106/10	Euclidean distance	NR	**All:** 2.06
Amsterdam (The Netherlands) Verburg et al. (49)	A and P	Frameless neuronavigation	7/99/5	Euclidean distance	NR	**All:** 3.5
Maastricht (The Netherlands) van der Loo et al. (50)	A and P	Conventional Frame-based	76/902/NR	Euclidean distance	**All:** 1.54	**All:** 2.93

*EP, Entry point error; NR, Not reported; TP, Target point error*.

##### SEEG planning, vascular avoidance, and vascular complications

Clinicians fear vascular complications, as hemostatic control is impossible given the percutaneous nature of the technique, and the consequences of intracranial bleeding can be ominous.

During SEEG planning, particular care must be taken to avoid vascular structures. A vessel-free safety radius of 2 mm is often considered around the planned trajectory. Angiography has remained the gold standard vascular examination for trajectory planning. With advances in MRI, CT, and computer-assisted fusion and planning techniques over the past two decades, some groups have drifted toward other means of vascular imaging for preoperative trajectory planning in order to reduce the length of the workflow. Intraoperative vascular imaging possibilities have also emerged, such as injected angio-CT with O-Arm (52). Whether these changes provide substantial advantages remains to be clearly determined.

In Table 3, we have summarized the vascular complications, SEEG technique, and type of vascular imagery used for referencing in the currently available literature on insular targeting with robot or non–robot-assisted techniques. Major bleeding complications have been defined as those causing death, needing surgical treatment, or leaving permanent sequelae. In a recent meta-analysis revising all sampling trajectories, hemorrhagic risk was estimated to be around 1.0%. The most frequent type of hemorrhagic complication was intraparenchymal hematoma followed by subdural and epidural hematomas. In comparison, infections, hardware-related complications, and permanent neurologic deficits were, respectively, estimated at 0.8, 0.4, and 0.6% (64).

**Table 3:** Overview of insular SEEG literature regarding vascular imaging reference and vascular complications.

**Epilepsy center/study period**	**Number of patients**	**Vascular imaging reference**	**Method**	**Insular trajectory**	**Direct vascular related complication^a^**
**Grenoble (France)**					
Afif et al. (30) *1998–2005*	30	Angiography	Frame-based Robot-assisted	Oblique	One major bleeding after electrode removal not related to insula
Blauwblomme et al. (53) *2001–2010*	17	Angiography	Frame-based Robot-assisted	Orthogonal Oblique	No vascular complications
Gras-Combes et al. (54) *2009–2013*	6	Angiography Angiography	Frame-based Robot-assisted Frameless robot-assisted	Orthogonal Oblique	No vascular complications
Abel et al. (36) *2013–2017 < 2013*	17 18	Angiography Angiography	Frameless Robot-assisted vs. Frame-based robot-assisted	Orthogonal Oblique	4 minor bleedings not related to insula 2 minor bleeding not related to insula
**Lyon (France)**					
Isnard et al. (12) *1996–2001*	50	Angiography	Conventional Frame-based	Orthogonal	NR
Bourdillon et al. (55) *1995–2015*	459	Angiography	Conventional Frame-based and Frame-based robot-assisted	Orthogonal	7 major bleedings not related to insula with conventional frame-based method (1 death)
Bourdillon et al. (32) *2012–2015*	100	Angiography with O-arm	Conventional frame-based method and Frame-based robot-assisted	Orthogonal	No vascular complications
**Fondation Rotschild, Paris (France)**					
Dorfmüller et al. (56) Dylgjeri et al. (57) *2009–2012*	19 10	MRI Gado	Frameless robot-assisted	Orthogonal Oblique	No vascular complications
**Pitié-Salpétrière, Paris (France)**					
Mathon et al. (58) *1991–2014*	157	MRI Gado (+ TOF Gado)	Conventional Frame-based	NR	2 minor bleedings 3 major bleedings Not related to insula 1 stroke of the insula (temporal electrode insertion)
**Montpellier (France)**					
Gil Robles et al. (28) *< 2009*	9	MRI Gado	Conventional Frame-based	Parasagittal posterior	No vascular complications
**Strasbourg (France)**					
Ollivier et al. 62 *2010–2016*	66	MRI Gado	Frameless Robot-assisted	Oblique	8 minor bleedings 1 major bleeding (Not specified if insular trajectory involved)
**Nancy (France)**					
Salado et al. (29) *2008–2016*	99	MRI Gado (double injection)	Conventional frame-based	Orthogonal (97.3%) Oblique (2.7%)	6 minor bleedings 1 major bleeding (Not related to insula)
**Rouen (France)**					
Gilard et al. (48) * <2016*	10	CTA	Conventional frame-based	NR	No vascular complications
**Milan (Italy)**					
Cardinale et al. (59) *1996–2018*	713	Angiography (+/- with O-arm)	Conventional frame-based Frame-based robot-assisted Frameless robot-assisted since 2016	NR	5 major bleedings with conventional frame-based method. 0 major bleeding with robot (Not specified if insular trajectory involved)
**Roma (Italy)**					
De Benedictis et al. (43) *2011–2016*	116	MRI Gado	Frameless robot-assisted	NR	No vascular complications
**Barcelona (Spain)**					
Candela-Canto et al. (39) *2016–2018*	14	CTA (bi-phasic injection)	Frameless robot-assisted	NR	1 major bleeding not related to insula
**Vienna (Austria)**					
Desai et al. (25) *2014–2015*	16	MRI Gado	Frameless robot-assisted	Oblique	No vascular complications
**Frankfurt (Germany)**					
Spyrantis et al. (35) *2012–2018*	19	MRI Gado	Frameless robot-assisted	NR	No vascular complications
**Sofia (Bulgary)**					
Minkin et al. (60) *2013-2015*	34	MRI Gado (double injection) and MRA	Conventional Frame-based	Orthogonal (75%) Oblique (25%)	1 minor bleeding
**Amsterdam (The Netherlands)**					
Verburg et al. (49)	7	MRI Gado	Frameless Neuronavigation	NR	1 major bleeding after electrode removal (Not specified if insular trajectory involved)
**Maastricht (The Netherlands)**					
van der Loo et al. (50) *2008–2016*	71	MRI Gado	Conventional Frame-based	Orthogonal Oblique	2 major bleedings 4 minor bleedings
**Great ormond street hospital, London (UK)**					
Sharma et al. (44) *2014–2017*	14 6	CTA	Frame-based robot-assisted Frameless Neuronavigation	NR	No vascular complications
**National hospital for neurology and neurosurgery, London (UK)**					
Nowell et al. (47) * <2014*	22	CTA and 3D phase contrast MRI	Frameless Neuronavigation	Oblique	1 minor bleeding (Not specified if insular trajectory involved)
**London (Canada)**					
Bottan et al. (31) *2017-2018*	41	MRI Gado (double injection)	Frame-based robot-assisted	Orthogonal (15.3%) Oblique (82.7%) Parasagittal (2%)	No vascular complications
**Cleveland (USA)**					
McGovern et al. (61) *2009–2017*	526	Angiography MRI Gado alone or + CTA	Conventional frame-based method and frame-based robot-assisted and frameless robot-assisted since 2013	Orthogonal Oblique	93 minor bleedings^b^ 12 major bleedings (9 transient deficit 2 permanent deficits 1 death)
Alomar et al. (62) 2009–2013	135 with insular trajectories	Angiography (27 patients) MRI Gado + CTA (108 patients)	Conventional frame-based method (27) Frameless robot-assisted (108)	Orthogonal (88.5%) Oblique (11.5%)	1 minor bleeding not related to insula
**Lebanon (USA)**					
Desai et al. (27) *2001–2009*	20	MRI Gado	Conventional frame-based method	Frontal oblique	No vascular complications
**Saint Louis (USA)**					
Miller et al. (63) *2016*	11	CTA	Frameless robot-assisted	NR	1 minor bleeding
**Munt Sinaï; New York (USA)**					
Iordanou et al. (45) * <2018*	25	MRI Gado	Frameless robot-assisted	Orthogonal Oblique	No vascular complications reported

*CTA, Computed tomographic angiography; Gado, Gadolinium; MRI, Magnetic resonance imaging; NR, Not reported; TOF, Time of flight*.

a *Major bleeding refer to potential fatal or permanent sequelae bleeding or the need for surgical removal*.

b *The authors described all form of bleedings including subarachnoid minimal bleedings always excluded from other reports*.

Interestingly, in the series from Milan (59), no major bleeding events were observed since robotic assistance was adopted, using a frameless technique. In the series from Cleveland (61), it appears that the adoption of robotic assistance has also had a beneficial effect, with a tendency for a lower hemorrhagic risk when comparing the technique to the conventional frame-based method. The Cleveland Group also described a higher hemorrhagic risk when MRI was used for vascular assessment compared to angiography, and they have currently added computed tomographic angiography (CTA) to their planning workflow, resulting in a decrease in hemorrhagic events (34).

In the consideration of vascular imaging for trajectory planning, another question arises. Should we also consider the smallest vessels as potential hazards? Are larger vessels more prone to be injured or more resistant to traction?

In studies made on susceptibility weighted imaging (SWI) or digital subtraction angiography (DSA), it appears that trajectories at the vicinity of vessels of a caliber <1.5 mm in the brain parenchyma (not at the cortical entry) could not be hazardous (65). Other authors suggest that SWI may overestimate the vasculature and thus limit the number of trajectories without influencing the bleeding incidence (66). The topic is still a matter of debate with some authors considering that “the better we can see the enemy, the better we can plan to avoid it” (67).

In the currently available literature, insular trajectories do not seem to be associated with higher hemorrhagic complications, at least for orthogonal trajectories (55). In our experience, neither oblique nor orthogonal trajectories seem to be associated with an overexposure risk of bleeding.

##### Outcomes after insular SEEG

The main challenge in interpreting insular SEEG tracings consists in differentiating primary insular involvement from early secondary involvement. In the case of temporo-insular epilepsy (temporal plus epilepsy—TPE), non-recognition of primary insular involvement can lead to resective surgery failure (7, 68). On the other hand, resection of the insula, when its involvement is a secondary event, does not improve resective surgery outcomes but exposes the patient to unnecessary surgical risks (53). The utility of SEEG is thus self-evident in these complex situations.

Isnard et al. (12) described insular or operculo-insular epilepsy in 10% of the patients from their cohort. In one patient (2%), the EZ was temporo-insular (TPE). In the vast majority of their series (86% of patients), the insular involvement was secondary to a temporal onset zone. Afif et al. (30) similarly showed 17% of primary insular epileptic involvement and 50% of secondary insular involvement. Alomar et al. (62) found 17% of primary involvement but only 0.37% of secondary involvement. Desai et al. (27) described 10% of primary and 25% of secondary insular involvement. Salado et al. (29) showed 41% of primary and 22% of secondary insular.

Whether these differences emerge from a high variability of epileptic syndromes, a topographical or genetic fingerprint or simply differences in SEEG sampling strategies and techniques remains unclear. This should be resolved as the amount of available data increase.

In the papers previously cited, resective surgery was the most frequently proposed therapeutic option, except when eloquent areas were in the EZ or when bilateral epilepsy was the culprit.

In cases where resective surgery is functionally impossible, vagal nerve stimulation can be proposed. An alternative approach has emerged with focal EZ ablation by thermal therapy. Laser-interstitial thermal therapy (LITT) has indeed been proposed by some authors (62, 69) with encouraging results. However, its availability across the world is still limited due to its cost, its highly complex technical platform, and its expertise requirement. In Europe, radio-frequency thermocoagulation (RF-TC) has been developed to address cases of non-resectable EZs, and sometimes as a primary treatment strategy when an extremely focal EZ is detected on SEEG.

##### Radio-frequency thermo-coagulation in the insula

Stereotactic lesion formation in epilepsy is not a novel approach, but the use of SEEG electrodes for RF-TC was published by Guénot et al. (70) in Lyon. They used this strategy in the insula in two patients with an 80% decrease in seizure events and no permanent adverse events (one patient suffered from transient oral paresthesia).

In our experience, based on 23 patients, seven RF-TC procedures were performed on the insula. Two patients had beneficial effects from the procedure (71). In the biggest insular RFTC-dedicated series published by Saint Anne's group (Paris), 89% of their 19 patients were good responders with 10 Engels's class I, 4 class II, and 3 class III patients (72). RFTC was performed as a separate procedure after SEEG. Transient postoperative deficits were observed in 42% (eight patients) with mild hemiparesis, hypoesthesia, dysgeusia, or dysarthria. Only one remained in a permanent mild dysarthria.

Finally, RF-TC can be used alone or in combination with insular or extra-insular corticectomy (29) and can be repeated in case of relapse (73) at the cost of a new lead implantation. Of note, performing RF-TC does not contraindicate subsequent surgery and can even be used as a strong predictive factor of postsurgical outcome (74).

## Conclusion

The potential of preoperative SEEG exploration for planning effective and safe epilepsy surgeries has been recognized and established over the past decades. The technique has proven to be particularly useful when the insula needs to be explored as symptoms of insular epilepsy are harder to recognize or detect on a standard EEG. With the advances in robotic-assisted surgery, SEEG techniques have been simplified, and their accuracy has increased, thus diminishing the intervention risks and time constraints.

The future of the technique will most probably rely on computer-assisted planning strategies in order to reduce the time needed for trajectory planning and will probably employ artificial intelligence-based software (75). Further developments could come from accuracy registration verification in the operating room, with real-time augmented reality allowing an increase in accuracy (76).

While the dawn of SEEG seems to have passed and the technique has established itself as an essential tool for surgical planning in epilepsy surgery, the future of the technique appears brighter than ever, as robotics and software automatization aid neurosurgeons in this complex task.

## Author Contributions

AD wrote the manuscript and built the figures. JZ-J revised the manuscript and co-built the figures. DH, A-SJ-C, PK, and LM revised the manuscript. ED co-wrote the manuscript. SC co-wrote the manuscript and provided final manuscript revision. All authors contributed to the article and approved the submitted version.

## Conflict of Interest

SC has worked as a consultant for Zimmer Biomet. The remaining authors declare that the research was conducted in the absence of any commercial or financial relationships that could be construed as a potential conflict of interest.
